# Supplementary material for: An open-source framework for large-scale, flexible evaluation of biomedical text mining systems
Source: J Biomed Discov Collab. 2008 Jan 29;3:1. doi: 10.1186/1747-5333-3-1 (PMC2276192; doi:10.1186/1747-5333-3-1)
Supplement: Additional file 1 — Evaluation platform source code. The additional file contains the software evaluation framework discussed in this paper. The most current release can be downloaded from SourceForge.net [1]. See the accompanying README file for instructions on installation and use. Also included in the distribution is a collection of UIMA wrappers for some commonly used BioNLP tools and annotated corpora. [file 1747-5333-3-1-S1.zip › bionlp_uima_repository-1.0.1/docs/api/edu/uchsc/ccp/util/nlp/annotation/TextAnnotation.html]

TextAnnotation (Center for Computational Pharmacology Util/UIMA API)


|  |  |  |
| --- | --- | --- |
| **Overview** \| **Package** \| **Class** \| **Tree** \| **Deprecated** \| **Index** \| **Help** | |  |
| **PREV CLASS**   NEXT CLASS | **FRAMES**    **NO FRAMES**     **All Classes** |  |
| SUMMARY: NESTED \| FIELD \| CONSTR \| METHOD | DETAIL: FIELD \| CONSTR \| METHOD |  |


---


## edu.uchsc.ccp.util.nlp.annotation Class TextAnnotation

```
java.lang.Object
  edu.uchsc.ccp.util.nlp.annotation.TextAnnotation
```

**All Implemented Interfaces:**: Comparable

---

``` public class TextAnnotation extends Object implements Comparable ```

This class is a generalized representation of a text annotation.

**Author:**
:   William A. Baumgartner, Jr.

---

**Constructor Summary**

`TextAnnotation()`
  
          **Deprecated.**

`TextAnnotation(int beginIndex,
int endIndex,
String coveredText,
Annotator annotator,
AnnotationSet annotationSet,
int annotationID,
int documentCollectionID,
String documentID,
int documentSectionID,
ClassMention classMention)`


| **Method Summary** | |
| --- | --- |
| `void` | `addAnnotationSet(AnnotationSet annotationSet)`             Add an annotation set |
| `void` | `addSpan(Span span)`             Add a span to this TextAnnotation. |
| `int` | `compareTo(Object textAnnotationToCompare)` |
| `boolean` | `equals(Object textAnnotationToEquate)`             The default equals() method requires exact span match, as well as identical class mention match to return true. |
| `Span` | `getAggregateSpan()`             Returns the total span of this annotation (i.e. |
| `int` | `getAnnotationID()`             Get the annotation ID |
| `Set<Integer>` | `getAnnotationSetIDs()`             Return a set containing the AnnotationSet IDs for which this TextAnnotation is a member. |
| `Set<AnnotationSet>` | `getAnnotationSets()`             Get the annotation sets |
| `int` | `getAnnotationSpanEnd()`             Return the end index of the last span (spans are stored in order) |
| `int` | `getAnnotationSpanStart()`             Return the start index of the first span (spans are stored in order) |
| `Annotator` | `getAnnotator()`             Set the annotator |
| `int` | `getAnnotatorID()`             This method returns the corresponding annotator ID |
| `ClassMention` | `getClassMention()` |
| `String` | `getCoveredText()`             Return the text that is "covered" by this annotation |
| `int` | `getDocumentCollectionID()`             Get the document collection ID |
| `String` | `getDocumentID()`             Get the document ID |
| `String` | `getDocumentLevelSingleLineRepresentation(boolean deep)`             Same a single line representation, however no span, covered text, or annotation meta info, is shown.. |
| `int` | `getDocumentSectionID()`             Get the document section ID |
| `String` | `getHashKey()`             **Deprecated.** |
| `String` | `getSingleLineRepresentation(boolean showDocID, boolean deep)`             return a String which represents this annotation, including all class mentions and any associated annotations NOTE: ANY CHANGES HERE MUST BE MIRRORED IN Knowtator\_Util.getSingleLineRepresentationOfAnnotation() and probably UIMA\_Util |
| `List<Span>` | `getSpans()`             Get the ordered list of spans for this TextAnnotation |
| `int` | `hashCode()` |
| `boolean` | `isMemberOfAnnotationSet(int annotationSetID)`             Return true if this TextAnnotation is a member of the inputted annotation set, false otherwise. |
| `int` | `length()`             Return the length of this TextAnnotation. |
| `boolean` | `overlaps(TextAnnotation ta)`             returns true if the total span of this TextAnnotation overlaps with the total span of the input TextAnnotation |
| `void` | `printAnnotation(PrintStream ps)`             Output a representation of this TextAnnotation to the input PrintStream |
| `void` | `printAnnotationOnOneLine(PrintStream ps)`             simple output on one line.. |
| `void` | `setAnnotationID(int annotationID)`             Set the annotation ID |
| `void` | `setAnnotationSets(Set<AnnotationSet> annotationSets)`             Set the AnnotationSet membership for this TextAnnotation |
| `void` | `setAnnotationSpanEnd(int spanEnd)`             Set the end index for the last annotation span |
| `void` | `setAnnotationSpanStart(int spanStart)`             Sets the start index for the first annotation span |
| `void` | `setAnnotator(Annotator annotator)`             Get the annotator |
| `void` | `setAnnotatorID(int annotatorID)`             **Deprecated.** |
| `void` | `setClassMention(ClassMention classMention)`             When setting the classmention, the a reference to the text annotation is automatically created inside the classMention object. |
| `void` | `setCoveredText(String coveredText)`             Set the text that is "covered" by this annotation |
| `void` | `setDocumentCollectionID(int documentCollectionID)`             Set the document collection ID |
| `void` | `setDocumentID(String documentID)`             Set the document ID |
| `void` | `setDocumentSectionID(int documentSectionID)`             Set the document section ID |
| `void` | `setSpan(Span span)`             Set the annotation span to be equal to the single input span. |
| `void` | `setSpans(List<Span> spans)`             Set the span list equal to the inputted span list |

**Methods inherited from class java.lang.Object**

`clone, finalize, getClass, notify, notifyAll, toString, wait, wait, wait`

**Constructor Detail**

### TextAnnotation

```
public TextAnnotation(int beginIndex,
                      int endIndex,
                      String coveredText,
                      Annotator annotator,
                      AnnotationSet annotationSet,
                      int annotationID,
                      int documentCollectionID,
                      String documentID,
                      int documentSectionID,
                      ClassMention classMention)
```

**Parameters:**: `beginIndex` -: `endIndex` -: `coveredText` -: `annotator` -: `annotationSet` -: `annotationID` -: `documentCollectionID` -: `documentID` -: `documentSectionID` -: `classMention` -

---


### TextAnnotation

```
@Deprecated
public TextAnnotation()
```

:   **Deprecated.**

    :   Default Constructor - creates default TextAnnotation - I would like to phase this out, therefore the Deprecated flag.
        Default values: beginIndex = -1   
        endIndex = -1   
        coveredText = empty  
        String annotatorID = -1   
        annotationSetID = -1   
        annotationID = -1   
        documentCollectionID = -1   
        documentID = -1   
        documentSectionID = -1   
        classMentionMap = empty HashMap


**Method Detail**

### getAnnotatorID

```
public int getAnnotatorID()
```

:   This method returns the corresponding annotator ID

    :   **Returns:**: annotator ID

---


### setAnnotatorID

```
@Deprecated
public void setAnnotatorID(int annotatorID)
```

:   **Deprecated.**

    :   This method sets the annotator ID

        :   **Parameters:**: `annotatorID` -

---


### getAnnotator

```
public Annotator getAnnotator()
```

:   Set the annotator

    :   **Returns:**

---


### setAnnotator

```
public void setAnnotator(Annotator annotator)
```

:   Get the annotator

    :   **Parameters:**: `annotator` -

---


### getAnnotationSets

```
public Set<AnnotationSet> getAnnotationSets()
```

:   Get the annotation sets

    :   **Returns:**

---


### addAnnotationSet

```
public void addAnnotationSet(AnnotationSet annotationSet)
```

:   Add an annotation set

    :   **Parameters:**: `annotationSet` -

---


### getAnnotationSetIDs

```
public Set<Integer> getAnnotationSetIDs()
```

:   Return a set containing the AnnotationSet IDs for which this TextAnnotation is a member.

    :   **Returns:**

---


### setAnnotationSets

```
public void setAnnotationSets(Set<AnnotationSet> annotationSets)
```

:   Set the AnnotationSet membership for this TextAnnotation

    :   **Parameters:**: `annotationSets` -

---


### isMemberOfAnnotationSet

```
public boolean isMemberOfAnnotationSet(int annotationSetID)
```

:   Return true if this TextAnnotation is a member of the inputted annotation set, false otherwise.

    :   **Parameters:**: `annotationSetID` - **Returns:**

---


### getAnnotationID

```
public int getAnnotationID()
```

:   Get the annotation ID

    :   **Returns:**

---


### setAnnotationID

```
public void setAnnotationID(int annotationID)
```

:   Set the annotation ID

    :   **Parameters:**: `annotationID` -

---


### getDocumentCollectionID

```
public int getDocumentCollectionID()
```

:   Get the document collection ID

    :   **Returns:**

---


### setDocumentCollectionID

```
public void setDocumentCollectionID(int documentCollectionID)
```

:   Set the document collection ID

    :   **Parameters:**: `documentCollectionID` -

---


### getDocumentID

```
public String getDocumentID()
```

:   Get the document ID

    :   **Returns:**

---


### setDocumentID

```
public void setDocumentID(String documentID)
```

:   Set the document ID

    :   **Parameters:**: `documentID` -

---


### getDocumentSectionID

```
public int getDocumentSectionID()
```

:   Get the document section ID

    :   **Returns:**

---


### setDocumentSectionID

```
public void setDocumentSectionID(int documentSectionID)
```

:   Set the document section ID

    :   **Parameters:**: `documentSectionID` -

---


### getCoveredText

```
public String getCoveredText()
```

:   Return the text that is "covered" by this annotation

---


### setCoveredText

```
public void setCoveredText(String coveredText)
```

:   Set the text that is "covered" by this annotation

    :   **Parameters:**: `coveredText` -

---


### getSpans

```
public List<Span> getSpans()
```

:   Get the ordered list of spans for this TextAnnotation

    :   **Returns:**

---


### setSpans

```
public void setSpans(List<Span> spans)
```

:   Set the span list equal to the inputted span list

    :   **Parameters:**: `spans` -

---


### setSpan

```
public void setSpan(Span span)
```

:   Set the annotation span to be equal to the single input span.

    :   **Parameters:**: `span` -

---


### addSpan

```
public void addSpan(Span span)
```

:   Add a span to this TextAnnotation. The span will be placed in the proper position in the ordered span list.

    :   **Parameters:**: `span` -

---


### getAnnotationSpanStart

```
public int getAnnotationSpanStart()
```

:   Return the start index of the first span (spans are stored in order)

    :   **Returns:**

---


### setAnnotationSpanStart

```
public void setAnnotationSpanStart(int spanStart)
```

:   Sets the start index for the first annotation span

    :   **Parameters:**: `spanStart` -

---


### getAnnotationSpanEnd

```
public int getAnnotationSpanEnd()
```

:   Return the end index of the last span (spans are stored in order)

---


### setAnnotationSpanEnd

```
public void setAnnotationSpanEnd(int spanEnd)
```

:   Set the end index for the last annotation span

    :   **Parameters:**: `spanEnd` -

---


### getClassMention

```
public ClassMention getClassMention()
```

---


### setClassMention

```
public void setClassMention(ClassMention classMention)
```

:   When setting the classmention, the a reference to the text annotation is automatically created inside the classMention object.

    :   **Parameters:**: `classMention` -

---


### getHashKey

```
@Deprecated
public String getHashKey()
```

:   **Deprecated.**

    :   Return a String that can be used as a hashkey for this TextAnnotation - I wonder if this should be deprecated?!

        :   **Returns:**

---


### printAnnotation

```
public void printAnnotation(PrintStream ps)
```

:   Output a representation of this TextAnnotation to the input PrintStream

    :   **Parameters:**: `ps` -

---


### getSingleLineRepresentation

```
public String getSingleLineRepresentation(boolean showDocID,
                                          boolean deep)
```

:   return a String which represents this annotation, including all class mentions and any associated annotations
    NOTE: ANY CHANGES HERE MUST BE MIRRORED IN Knowtator\_Util.getSingleLineRepresentationOfAnnotation()
    and probably UIMA\_Util

    :   **Returns:**

---


### getDocumentLevelSingleLineRepresentation

```
public String getDocumentLevelSingleLineRepresentation(boolean deep)
```

:   Same a single line representation, however no span, covered text, or annotation meta info, is shown.. just the class mention

    :   **Parameters:**: `showDocID` -: `deep` - **Returns:**

---


### printAnnotationOnOneLine

```
public void printAnnotationOnOneLine(PrintStream ps)
```

:   simple output on one line.. will not print complex slot mentions

    :   **Parameters:**: `ps` -

---


### compareTo

```
public int compareTo(Object textAnnotationToCompare)
```

:   **Specified by:**: `compareTo` in interface `Comparable`

---


### equals

```
public boolean equals(Object textAnnotationToEquate)
```

:   The default equals() method requires exact span match, as well as identical class mention match to return true.

    :   **Overrides:**: `equals` in class `Object`

---


### getAggregateSpan

```
public Span getAggregateSpan()
```

:   Returns the total span of this annotation (i.e. from the start of the first span to the end of the last span)

---


### overlaps

```
public boolean overlaps(TextAnnotation ta)
```

:   returns true if the total span of this TextAnnotation overlaps with the total span of the input TextAnnotation

    :   **Returns:**

---


### hashCode

```
public int hashCode()
```

:   **Overrides:**: `hashCode` in class `Object`

---


### length

```
public int length()
```

:   Return the length of this TextAnnotation. This method returns the length of the aggregate span.

    :   **Returns:**


---


|  |  |  |
| --- | --- | --- |
| **Overview** \| **Package** \| **Class** \| **Tree** \| **Deprecated** \| **Index** \| **Help** | |  |
| **PREV CLASS**   NEXT CLASS | **FRAMES**    **NO FRAMES**     **All Classes** |  |
| SUMMARY: NESTED \| FIELD \| CONSTR \| METHOD | DETAIL: FIELD \| CONSTR \| METHOD |  |


---
